# Distinct polymer physics principles govern chromatin dynamics in mouse and *Drosophila* topological domains

**DOI:** 10.1186/s12864-015-1786-8

**Published:** 2015-08-15

**Authors:** Vuthy Ea, Tom Sexton, Thierry Gostan, Laurie Herviou, Marie-Odile Baudement, Yunzhe Zhang, Soizik Berlivet, Marie-Noëlle Le Lay-Taha, Guy Cathala, Annick Lesne, Jean-Marc Victor, Yuhong Fan, Giacomo Cavalli, Thierry Forné

**Affiliations:** Institut de Génétique Moléculaire de Montpellier, UMR5535, CNRS, Université de Montpellier, 1919 Route de Mende, 34293 Montpellier, Cedex 5 France; Institut de Génétique Humaine, UPR 1142, CNRS, Montpellier, France; School of Biology and the Petit Institute for Bioengineering and Bioscience, Georgia Institute of Technology, Atlanta, Georgia USA; CNRS GDR 3536 UPMC, Sorbonne universités, Paris, France; Laboratoire de Physique de la Matière Condensée, CNRS UMR 7600, UPMC, Sorbonne universités, Paris, France

**Keywords:** Chromatin dynamics, Polymer models, Topological domains, Epigenetics, H1 histone

## Abstract

**Background:**

In higher eukaryotes, the genome is partitioned into large "Topologically Associating Domains" (TADs) in which the chromatin displays favoured long-range contacts. While a crumpled/fractal globule organization has received experimental supports at higher-order levels, the organization principles that govern chromatin dynamics within these TADs remain unclear. Using simple polymer models, we previously showed that, in mouse liver cells, gene-rich domains tend to adopt a statistical helix shape when no significant locus-specific interaction takes place.

**Results:**

Here, we use data from diverse 3C-derived methods to explore chromatin dynamics within mouse and *Drosophila* TADs. In mouse Embryonic Stem Cells (mESC), that possess large TADs (median size of 840 kb), we show that the statistical helix model, but not globule models, is relevant not only in gene-rich TADs, but also in gene-poor and gene-desert TADs. Interestingly, this statistical helix organization is considerably relaxed in mESC compared to liver cells, indicating that the impact of the constraints responsible for this organization is weaker in pluripotent cells. Finally, depletion of histone H1 in mESC alters local chromatin flexibility but not the statistical helix organization. In *Drosophila*, which possesses TADs of smaller sizes (median size of 70 kb), we show that, while chromatin compaction and flexibility are finely tuned according to the epigenetic landscape, chromatin dynamics within TADs is generally compatible with an unconstrained polymer configuration.

**Conclusions:**

Models issued from polymer physics can accurately describe the organization principles governing chromatin dynamics in both mouse and *Drosophila* TADs. However, constraints applied on this dynamics within mammalian TADs have a peculiar impact resulting in a statistical helix organization.

**Electronic supplementary material:**

The online version of this article (doi:10.1186/s12864-015-1786-8) contains supplementary material, which is available to authorized users.

## Background

During the last decade, the advent of Chromosome Conformation Capture (3C) [1] and its derived technologies (4C, 5C, Hi-C) [2] allowed to explore genome organization with unprecedented resolution and accuracy. By capturing all chromatin contacts present at a given time in their physiological nuclear context, and then by averaging these events over several millions of cells, the quantitative 3C method [3] allows to access the relative contact frequencies between chromatin segments *in vivo*. This feature is key to understanding chromatin dynamics *in vivo* because it depends not only on fundamental biophysical parameters of the chromatin (such as compaction and stiffness) that determine its local organization at the nucleosomal scale, but also on constraints that impact its higher-order/supranucleosomal organization. These latter constraints can result either from nuclear determinants that organize chromatin at higher scales (“top-down” constraints) or from some intrinsic locus-specific components of the chromatin that are controlling genomic functions, like epigenetic modifications or the binding of specific factors (“bottom-up” constraints) [4].

Hi-C approaches (that combine 3C assays with high-throughput sequencing) provided genome-wide profiling of contact frequencies in the yeast (*Saccharomyces cerevisiae*) [5], fly (*Drosophila melanogaster*) [6], mouse (*Mus musculus domesticus*) [7] and human [8, 9] genomes. While these data confirmed that higher-order chromatin dynamics appears to be globally unconstrained in yeast, they showed that this organization level is constrained in higher eukaryotes where the chromatin is compartmentalized into chromosomal territories that are themselves further partitioned into the so-called “Topologically Associating Domains” (TADs) [10] or contact domains [9]. TADs and contact domains are defined as chromosomal sub-compartments that display preferential contacts in *cis*. However, they are restricted to interphase cells and disappear in mitotic chromosomes [11], to be re-acquired in the early G1 phase [12]. They are physically delimited by borders that are gene-rich regions enriched in specific factors like the insulator protein CTCF [7, 9, 13, 14]. Noticeably, the location of TAD borders appears to be quite stable across cell types. It is commonly accepted that, within TADs, chromatin is organized into chromatin loops, via locus-specific interactions, and that this organization is tightly related to genome function [9, 15–17]. It has recently been evidenced that such interactions occur in the context of fluctuating structures rather than being stable loops [18], and we previously showed that, in the absence of strong long-range locus-specific interactions, this underlying dynamics of the chromatin undergo constraints in gene-rich regions resulting in modulated contact frequencies over large genomic distances [4]. While the involvement of locus-specific factors in chromatin-loop formation, within TADs, is now well established [9], the physical properties that govern the underlying chromatin dynamics at that scale remains unknown.

Here, using quantitative 3C experiments, we report that the modulation of contact frequencies previously described in liver cells [4] is also present in pluripotent mouse Embryonic Stem Cells (mESC), not only in gene-rich TADs, but also in gene-poor and gene-desert domains. Therefore, the constraints that affect higher-order chromatin dynamics in mammals appear to widely affect TADs in diverse genomic contexts. We show that the equilibrium/crumpled globule models do not reproduce chromatin dynamics within mammalian TADs. In contrast, models derived from polymer physics can accurately describe chromatin dynamics at that scale in both mouse and *Drosophila* TADs. In the mouse, we found that chromatin dynamics is less constrained in ESC than in liver cells, and that this constraint is also strongly attenuated in a TAD spanning a gene-desert compared to gene-poor or gene-rich TADs. In *Drosophila melanogaster*, using Hi-C data obtained from embryos, we show that, on a local scale, chromatin dynamics is finely tuned according to the epigenetic landscape: the nucleofilament is less compact and more flexible in active than in heterochromatic domains. However, in contrast to mammals, the higher-order chromatin dynamics in *Drosophila* appears largely unconstrained.

## Results

To explore the influence of the genomic context on chromatin dynamics, we first investigated mouse ESC, for which TADs have been finely defined [7]. We focused on three types of domains: five gene-rich TADs, two gene-poor TADs [19] and one gene-desert TAD (Additional file 1a and b). The regions investigated in the two gene-poor TADs (Additional file 1b) are devoid of any known genes or putative regulatory elements, and their homozygous deletion in mouse results in fully viable pups, with no obvious alteration [19]. These TADs actually do contain several genes, but the closest from the regions analysed are located around 300 kb away. In contrast, the gene-desert TAD is containing a single gene located more than 1.5 Mb away from the region analysed (Additional file 1a).

### Equilibrium/crumpled globule models do not reproduce chromatin dynamics within mammalian TADs

Equilibrium and crumpled/fractal globule models, have been developed to describe chromatin dynamics *in vivo*. It was shown that, when one looks at decreasing contact frequencies as a function of increasing genomic distances in a Log-Log plot, the equilibrium globule model follows a power-law scaling associated to a slope of −3/2 over two orders of magnitude while the crumpled globule model has a slope of −1 [20]. Using Hi-C data, it was shown that crumpled globule features are characteristic of chromatin dynamics above 1 Mb (chromosome territory/inter-TADs dynamics) but that they may not be valid for separation distances shorter than 100 kb [8].

**Fig. 1:**
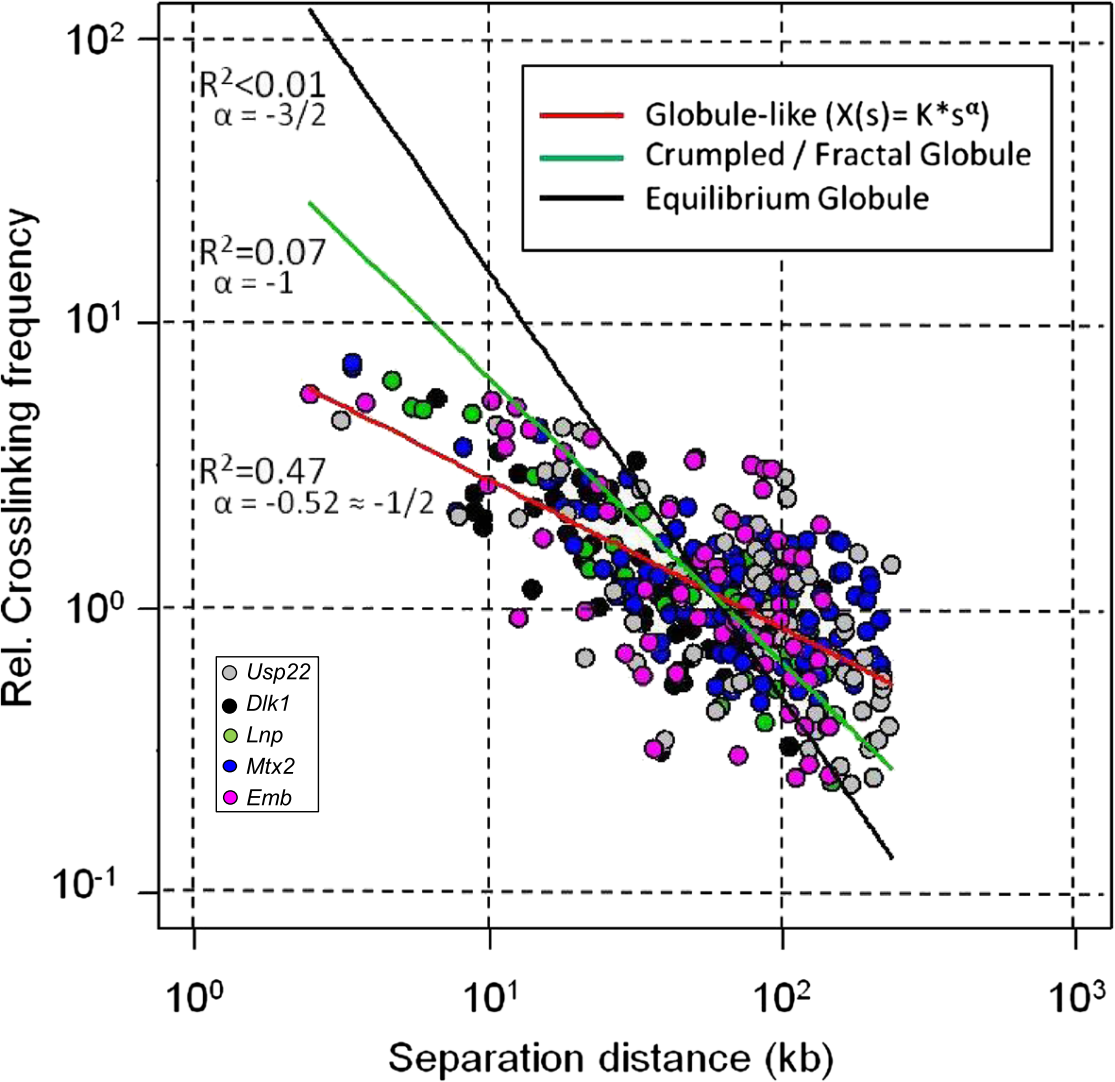
Fitting globule models to contact frequencies quantified in mESC. Experimental 3C-qPCR data obtained for wt mESC in gene-rich TADs (Fig. 2) have been displayed into a Log-Log plot and globule models were fitted to the following power-law: *X(s)* = k*s ^*α*^ (adapted from Eq. 6 and Eq. 9 from ref. [20]), where *X*(*s*) is the cross-linking frequency, *s* (in kb) is the site separation along the genome, *K* is representing the efficiency of cross-linking and the exponent *α* is the slope associated to this power-law. Best-fits (using the nls object of the R software) show that the slope associated to our experimental data (red line) is approximately *α* = −1/2 (−0.52) with a correlation coefficient *R* ^*2*^ = 0.47, while correlation coefficients associated to the equilibrium (*α* = −3/2) (black line) or crumpled globules (*α* = −1) (green line) are much lower

To assess whether such organization principles apply to chromatin dynamics within TADs, we thus performed quantitative 3C experiments in the different TADs described above and, using Log-Log plots, we showed that gene-rich, as well as gene-poor and gene-desert TADs display slopes superior to −1 (−0.60 to −0.48) (Fig. 1) which are incompatible with the equilibrium or crumpled globule models. Therefore, neither the equilibrium nor the crumpled globule models accurately reproduce chromatin dynamics within mammalian TADs.

### Chromatin dynamics is less constrained in pluripotent mESC than in liver cells

We then fitted our data to two models, derived from polymer physics, that were previously used to describe chromatin dynamics in the yeast *Saccharomyces cerevisiae* [1, 21] and in mammals [4]. The first model [see equations (eqs.) 1 and 2 in *Methods*] provides measurements of three key parameters of local chromatin dynamics (nucleosomal scale): *K* reflects features of the experimental setting (mainly cross-linking efficiency); *L* is the length of a chromatin segment (in nm) containing 1 kb of genomic DNA, thus reflecting chromatin compaction (in nm/kb); *S* (the Kuhn’s statistical segment, in kb) is a measure of chromatin flexibility [1, 21]. The higher cross-linking efficiency, chromatin compaction and flexibility, the lower the values of parameters *K, L* and *S* will be. This model assumes that, at higher-order organization levels, chromatin does not undergo any special constraints. Therefore, we define it as “unconstrained chromatin” model. The second model is named “statistical helix” model. It provides measurements of the same parameters of local chromatin dynamics, but it also takes into account constraints that may impact chromatin dynamics at the higher-order level (supranucleosomal scale). In this model, the higher-order chromatin dynamics is described as if constraints imposed onto chromatin were folding, statistically, the chromatin into a helical shape that can be characterized by two parameters: its mean Diameter *(D*) (in nm) and its mean Pitch (*P*) (in nm) [eq.2] [4]. These two parameters are thus describing the presence of constraints that impact higher-order chromatin dynamics. The weaker the effects of the constraints, the less pronounced the parameters of the statistical helix will be (i.e. large diameter and/or large Pitch).

**Fig. 2:**
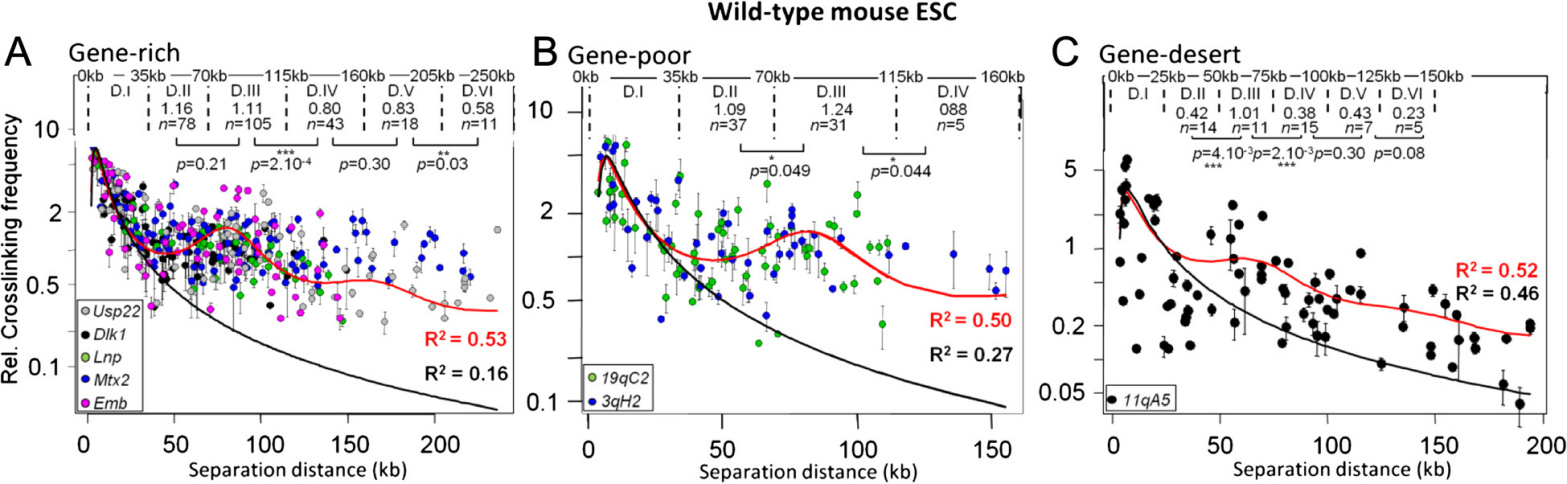
Fitting the statistical helix model to contact frequencies quantified in mESC. Quantitative 3C data were obtained from wild-type mouse ESC in five gene-rich TADs (**a**), two gene-poor TADs (**b**) and one gene-desert TAD (**c**) (see genomic maps in Additional file 1). For each type of TAD, data obtained from all the anchor primers used for each locus (Additional file 7) were compiled in a single graph (each locus is represented by a specific color). Error bars are standard error of the mean of three independent quantitative 3C assays each quantified at least in triplicate. Dashed lines delimit supranucleosomal domains that encompass separation distances where contact frequencies are alternatively lower and higher (see Methods). The graphs show the best fit analyses obtained with the unconstrained chromatin model [eqs. 1 and 2] (black curves) or the statistical helix model [eqs. 1 and 3] (red curves). Correlation coefficients (*R* ^2^) are indicated on the graphs. Best fit parameters, and the genomic distance contained within one statistical helix turn (*Sh* in kb), are given in the upper part of Table 1. For each supranucleosomal domains, the mean contact frequencies and the number (*n*) of experimental points are indicated on the graphs. *p*-values (Mann–Whitney *U*-test) account for the significance of the differences observed between the experimental means of two adjacent domains (double asterisks indicate a *p*-value < 0.05 and > 0.01 and triple asterisks a *p*-value < 0.01)

As previously found in mouse liver cells [4], gene-rich TADs display modulated contact frequencies and the statistical helix model [eqs.1 and 3] can be very well fitted to our experimental data while the unconstrained chromatin model [eqs.1 and 2] does not fit for site separation larger than 35 kb (Fig. 2a). This confirms that, in both liver cells and mESC, chromatin dynamics in gene-rich TADs undergoes constraints that can be described by polymer models as if, at the supranucleosomal scale, the chromatin was statistically folded into a helix.

However, close examination of best-fit parameters indicates that the statistical helix organization of the chromatin in gene-rich TADs is considerably more relaxed in mESC compared to liver cells (Table 1, compare first and second rows). The mean Pitch (*P*) of the statistical helix is 201 ± 13 nm in mESC while it is only 160 ± 9 nm in liver cells, and the mean diameter (*D*) is 255 ± 8 nm and 287 ± 5 nm respectively. Consequently, one turn of the statistical helix (*Sh*) contains 97 ± 1 kb of genomic DNA in liver cells while it encompasses only 85 ± 2 kb in mESC. Therefore, higher-order chromatin dynamics is less constrained in pluripotent mESC than in liver cells. Remarkably, this clear difference is not linked to local chromatin flexibility since the *S* parameter is identical (*S* = 2.7 ± 0.1 kb) in both cell types (Table 1, upper part). Finally, the values of the *K* parameter suggest that cross-linking efficiency is higher in liver cells than in mESC (Table 1, compare first and second rows).

**Table 1:** Fitting the statistical helix model to the relative contact frequencies observed in wild-type (upper part, rows 2–4) and triple KO (lower part, rows 5–7) mouse ES cells (mESCs)

	WT vs H1 TKO mESC	K *10 ^*3*^	*S* (kb)	<*D* > (nm)	<*P* > (nm)	*Sh* (kb)
1	mouse liver gene-rich	890 ± 70	2.7 ± 0.1	287 ± 5	160 ± 9	97 ± 1
2	WT mESC gene-rich (Fig. 2a)	1,070 ± 80	2.7 ± 0.1	**255 ± 8**	**201 ± 13**	**85 ± 2**
3	WT mESC gene-poor (Fig. 2b)	1,880 ± 360	**3.7 ± 0.3**	262 ± 18	213 ± 31	87 ± 5
4	WT mESC gene-desert (Fig. 2c)	1,380 ± 370	**3.8 ± 0.3**	208 ± 44	264 ± 77	**72 ± 13**
5	H1TKO gene-rich (Fig. 3a)	1,810 ± 140	**3.1 ± 0.1**	268 ± 8	230 ± 14	83 ± 2
6	H1TKO gene-poor (Fig. 3b)	2,270 ± 430	3.7 ± 0.3	269 ± 19	224 ± 30	83 ± 5
7	H1TKO gene-desert (Fig. 3c)	2,620 ± 840	3.9 ± 0.4	264 ± 105	380 ± 162	86 ± 28

Parameters obtained for mouse liver cells [4] are indicated for comparisons (row 1). Remarkable values are indicated in bold (see text)

### Effects of constraints on chromatin dynamics correlate with gene density in mESC TADs

Interestingly, inside both gene-poor and gene-desert TADs, chromatin also displayed modulated contact frequencies (see mean contact frequencies in Fig. 2b/c), indicating that the constraints that impact higher-order chromatin dynamics are present in all genomic contexts investigated. However, while the statistical helix model fits again better than the unconstrained chromatin model to gene-poor TAD data (Fig. 2b), both models could be equally well fitted to the gene-desert data (Fig. 2c), indicating that chromatin dynamics in this latter TAD is not subject to strong constraints. Indeed, the statistical helix is relaxed in gene-desert TADs since one helix turn contains only 72 kb of genomic DNA while it encompasses more than 85/87 kb in gene-rich or gene-poor TADs (Table 1, compare the fourth row with the second and third rows). Globally, these results indicate that the shape of the statistical helix is progressively more elongated as we go from gene-rich and gene-poor to gene-desert TADs approaching an unconstrained chromatin configuration. Therefore, while the constraints impacting chromatin dynamics can be detected in all genomic contexts investigated, their effects are clearly stronger in gene-rich and in gene-poor than in gene-desert TADs.

The models also show that, at the nucleosomal scale, the chromatin is much less flexible in gene-poor (*S* = 3.7 ± 0.3 kb) and gene-desert (*S* = 3.8 ± 0.3 kb) TADs than in gene-rich TADs (*S* = 2.7 ± 0.1 kb) (Table 1, compare the third and fourth rows with the second row). However, these changes in chromatin flexibility do not necessarily translate into changes in higher-order chromatin dynamics. Indeed, gene-poor and gene-desert TADs have similar flexibility but different statistical helix organization: one helix turn encompasses 85/87 kb of genomic DNA in gene-poor TADs but only 72 kb in the gene-desert TAD (Table 1, compare third and fourth rows). Conversely, gene-rich and gene-poor TADs have different chromatin flexibility but very similar statistical helix: Pitch (*P*) is around 200 nm, diameter (*D*) is about 250 nm and one helix turn encompasses 85/87 kb of genomic DNA (Table 1, compare second and third rows). Finally, as we noted above, the statistical helix in gene-rich TADs is in a much more open configuration in mESC than in liver while chromatin flexibility is identical in both cell types (Table 1, compare first and second rows). Therefore, the variations of the higher-order chromatin dynamics observed *in vivo* in different genomic contexts appear to be largely independent of chromatin flexibility.

### Histone H1 depletion alters chromatin flexibility but not statistical helix organization

**Fig. 3:**
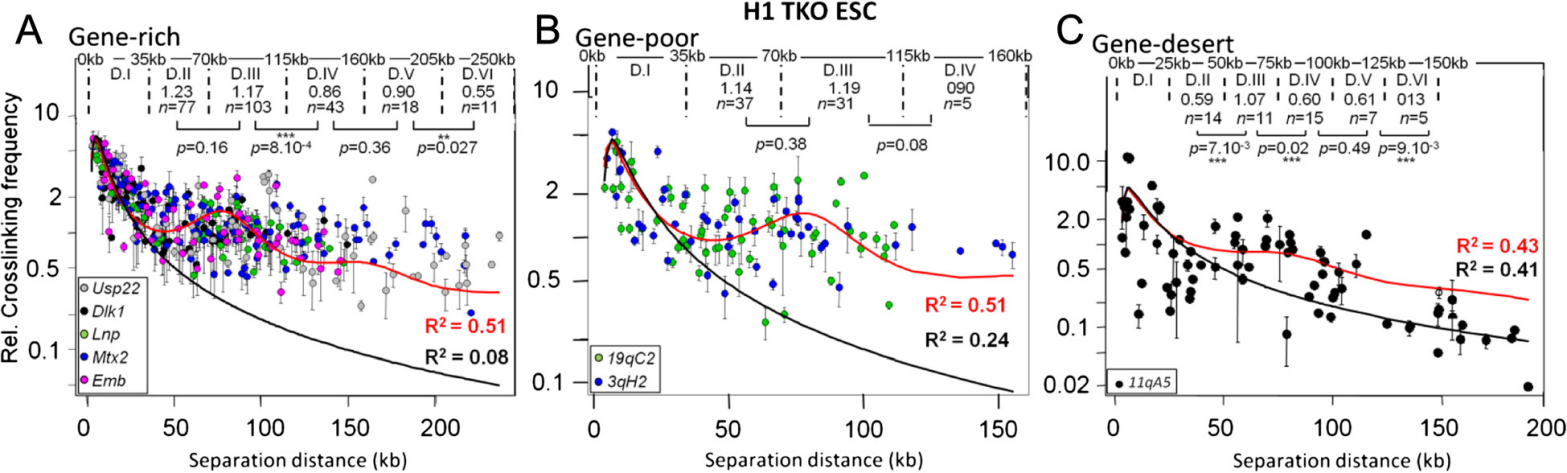
Fitting the statistical helix model to contact frequencies quantified in mouse H1 TKO ESC. Quantitative 3C data were obtained from mouse ESC that are Triple Knock-Out for Histone H1 genes (H1 TKO), for five gene-rich TADs (**a**), two gene-poor TADs (**b**) and one gene-desert TAD (**c**). The graphs show the best-fit analyses obtained with the unconstrained chromatin model [eqs. 1 and 2] (black curves) or the statistical helix model [eqs. 1 and 3] (red curves). The data (see Additional file 8) were analyzed and are depicted as described in the legend of Fig. 2. Best-fit parameters, and the genomic distance contained within one statistical helix turn (*Sh* in kb), are given in the lower part of Table 1

To ascertain that variations of chromatin flexibility do not necessarily impact higher-order chromatin dynamics, we performed quantitative 3C experiments in mESC that are Triple Knock-Out (H1 TKO) for histone H1 genes *H1c*, *H1d* and *H1e* [22]. Indeed, since it binds between nucleosomes, the linker histone H1 is thought to be a major factor regulating chromatin compaction and flexibility at the nucleosomal scale [23, 24], but a precise evaluation of its potential role for chromatin dynamics at the supranucleosomal scale is missing. Its depletion should thus allow us to assess whether altering chromatin stiffness will impact higher-order chromatin dynamics. Mice lacking the H1c, H1d and H1e die during embryonic development, but H1 TKO mESC lines can be established, which bear various chromatin structure changes [22]. Identical experiments as described above were thus performed in H1 TKO mESC (Fig. 3) and best-fit parameters of the statistical helix model were obtained for each category of TADs (Table 1, lower part).

In both gene-poor and gene-desert TADs (Fig. 3b and c respectively), where histone H1 density is very high [25], identical results were obtained in both H1 TKO and wild-type (WT) mESC (Table 1, compare third with sixth rows and fourth with seventh rows respectively). In these TADs, histone H1 depletion was apparently not sufficient to alter chromatin flexibility. One can note, however, that the values of the *K* parameter are higher in H1 TKO than in WT mESC (Table 1, compare third with sixth rows and fourth with seventh rows) indicating that cross-linking efficiency is lower upon partial histone H1 depletion.

In gene-rich TADs (Fig. 3a), where histone H1 density is lower [25] histone H1 depletion in mouse mESC resulted in a very significant decrease in chromatin flexibility compared to WT mouse mESC (*S* = 3.1 ± 0.1 kb and 2.7 ± 0.1 kb respectively) (Table 1, compare fifth and second rows). This result is in agreement with previous finding indicating that the stiffness of a disordered and poorly condensed chromatin fiber (as in H1 TKO mESC) is large, being directly influenced by the high stiffness of the embedded DNA stretch, while a more organized and condensed fiber (as in WT mESC) is far more flexible [26], provided that nucleosome stacking does not occur (as in gene-deserts where histone H1 density is very high) [27]. However, despite the significant decrease in chromatin flexibility observed in gene-rich TADs, the parameters of the statistical helix (diameter *D*, pitch *P*, DNA in helix turn *Sh*) were not significantly altered. The shape of the statistical helix tends to be slightly more elongated in H1 TKO mESC than in WT mESC, but this apparent tendency is not sufficiently strong to be considered as really significant. Therefore, the results presented in Fig. 3a demonstrate that altering chromatin flexibility at the nucleosomal scale in gene-rich TADs, where the statistical helix is prominent, does not necessarily impact significantly the higher-order chromatin organization of these regions.

This indicates that chromatin dynamics at the nucleosomal and supranucleosomal scales are somewhat uncoupled, suggesting that the constraints imposed on higher-order chromatin dynamics within TADs may not necessarily rely on intrinsic local features of the chromatin, like the presence of H1 linker histone or histone epigenetic modifications, which would affect its nucleosomal organization and oligonucleosome compaction [22]. Therefore, this raises the question of the role of the epigenetic landscapes on chromatin dynamics.

### Higher-order chromatin dynamics within *Drosophila* TADs is unconstrained

To investigate the influence of the epigenetic contexts on chromatin dynamics, we generated and used Hi-C data from the fly *Drosophila melanogaster* for which epigenetic domains have been extensively described [6]. The *Drosophila* genome is relatively small in size allowing ultra-high genomic resolution of chromatin contacts. Five billion paired-end Hi-C reads were obtained from late *Drosophila* embryos [28] and normalized Hi-C data were processed in order to produce thousands of “virtual 3C” profiles providing relative contact frequencies at 5 kb resolution throughout the *Drosophila* genome (see Methods).

To check whether some constraints impact chromatin dynamics in the *Drosophila*, we first focused our analyses on a subset of “virtual-3C” profiles spanning separation distances of at least 65 kb without crossing any TAD borders. Among the 2236 “virtual-3C” profiles that could be appropriately fitted to the unconstrained chromatin model [eqs. 1 and 2] (0 < R^2^ < 1), 66 % had a correlation coefficient (R^2^) above 0.5. This result indicates that the unconstrained chromatin model fits appropriately to most “virtual 3C” profiles and thus, in contrast to previous observation made in mammals [4] (Fig. 2), chromatin dynamics within *Drosophila* TADs appears as globally unconstrained, and hence non-helical, at the scale of several tens of kilo-bases.

### Local properties of *Drosophila* chromatin are finely tuned according to the epigenetic landscape

“Virtual 3C” generated were then classified according to chromosomal location and to the previously defined epigenomic domains (D1 to D4) [6]: D1 (“red chromatin”) corresponds to domains with “active” epigenetic marks, D2 (“black chromatin”) displays no specific epigenetic modifications, D3 (“blue chromatin”) is Polycomb (PcG) associated chromatin and D4 (“green chromatin”) is HP1/heterochromatin. Finally, for each “virtual 3C”, the unconstrained chromatin model was fitted and the three best-fit parameters were extracted (see Additional file 2 for representative examples). For each chromosome, statistical analyses of best-fit parameters were performed separately according to the epigenetic domains.

**Fig. 4:**
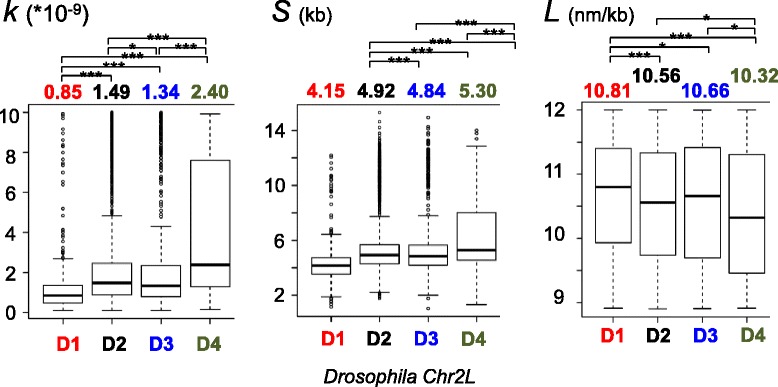
Epigenetic landscapes and chromatin dynamics of the *Drosophila* chromosome 2 L. “Virtual 3C”, obtained from Hi-C experiments in the *Drosophila*, were classified according to the four previously defined epigenetic domains (D1 to D4) [6]: D1 (“red chromatin”) corresponds to domains with “active” epigenetic marks, D2 (“black chromatin”) displays no specific epigenetic modifications, D3 (“blue chromatin”) is PcG associated chromatin and D4 (“green chromatin”) is HP1/heterochromatin. The unconstrained chromatin model [eqs.1 and 2] was then fitted and the three best-fit parameters (*K* = crosslinking efficiency; *L* = compaction; *S* = flexibility) were recovered from each “virtual 3C”. Statistical analyses of best-fit parameters were performed separately according to the epigenetic domains. Box-plots show the results obtained for each type of domains on the chromosome 2 L. Stars indicate statistically significant differences: single asterisk indicates a *p*-value < 0.05 and > 0.01, a double asterisk a *p*-value < 0.01 and > 0.001 and a triple asterisk a *p*-value < 0.001 (all *p*-values are given in Additional file 5). The number of best-fits (*n*) performed in each domain is as follows: D1: *n* = 990; D2: *n* = 2481; D3: *n* = 624; D4: *n* = 239). The results obtained from the other *Drosophila* chromosomes are given in Additional file 3

Box-plots in Fig. 4 show the results of statistical analyses of best-fit parameters obtained for chromosome 2 L. We found that “active” domains (D1, “red chromatin”) are less compact (median value of *L* parameter = 10.81 nm/kb), more efficiently cross-linked (median value of *K* parameter = 0.85) and more flexible (median value of *S* parameter = 4.15 kb) than the other domains (*L* = 10.56/10.66/10.32 nm/kb for D2/D3/D4 respectively while *K* = 1.49/1.34/2.40 and *S* = 4.92/4.84/5.30 kb for D2/D3/D4 respectively) (Fig. 4). As expected, we found that HP1/heterochromatin (D4) is much less flexible and more compact than any other type of chromatin. However, “black” (D2) and PcG (D3) chromatins have very similar flexibility and compaction, suggesting that PcG proteins do not significantly impact on local chromatin dynamics (Fig. 4). Identical results were found for all the other *Drosophila* chromosomes, except for the tiny chromosome 4, which displayed quite flexible and poorly compacted chromatin despite being entirely heterochromatic (Table 2) (full data are in Additional file 3. Additional file 5 gives Wilcox *p*-values of differences observed between the different epigenetic domains for parameters shown in Table 2). This finding is consistent with a recent work demonstrating that chromosome 4 displays distinct epigenetic profiles compared to both pericentric heterochromatin and euchromatic regions and that enrichment of HP1a on chromosome 4 genes creates an alternate chromatin structure which is critical for their regulation [29]. Globally, these experiments confirm that the epigenetic contexts influence significantly the local chromatin dynamics *in vivo*. However, quantitatively, their effects on chromatin compaction and flexibility appear as being quite limited. Indeed, the largest variations observed (between the “active” and HP1/heterochromatin domains) for chromatin compaction and flexibility are 10.76 to 9.99 nm/kb, i.e. about 7 %, on chromosome 2R, and 4.090 to 5.382 kb, i.e. about 24 %, on chromosome 3 L, respectively (Table 2). Therefore, the epigenetic landscape in the fly appears to be involved in fine-tuning the local chromatin dynamics.

**Table 2:** Fitting the unconstrained model on *Drosophila* Hi-C data^a^

Chromosome	Parameters	Active D1	Black D2	PcG D3	Centromeric D4
	K *10^9^	0.852	1.487	1.340	2.405
Chr2L	S (kb)	4.150	4.918	4.849	5.296
	L (nm/kb)	10.81	10.56	10.6	10,32
	K *10^9^	0.847	1.471	1.122	2.699
Chr2R	S (kb)	4.147	4.925	4.552	5.292
	L (nm/kb)	10.76	10.57	10.71	9.99
	K *10^9^	0.808	1.383	1.232	2.684
Chr3L	S (kb)	4.090	4.881	4.873	5.382
	L (nm/kb)	10.80	10.55	10.62	10.06
	K *10^9^	0.857	1.548	1.324	
Chr3R	S (kb)	4.132	4.95	4.773	
	L (nm/kb)	10.80	10.59	10.64	
	K *10^9^				1.303
Chr4	S (kb)				4.55
	L (nm/kb)				10.67

Median values of 3 best-fit parameters obtained on the autosomal chromosomes in each type of TADs

^a^Hi-C samples were prepared from unsexed flies, and therefore the X chromosome was not analysed since, in males, this chromosome undergoes dosage compensation that largely affects its epigenetic features

## Discussion

### Modulated contact frequencies, the statistical helix and their relevance for genome functions

Our work shows that, in the mouse, a modulation in contact frequency over large genomic distances can be detected in all the three genomic contexts investigated: gene-rich, gene-poor and gene-desert TADs. This demonstrates that the constraints responsible for the emergence of the statistical helix apply widely to the mammalian genome (Fig. 2; Table 1, upper part). However, their effects on higher-order chromatin dynamics are progressively attenuated as we shift from gene-rich and gene-poor to gene-desert TADs where, in this latter case, an unconstrained polymer model can be fitted appropriately to contact frequency data (Fig. 2; Table 1, upper part). This situation is reminiscent to experiments performed in the yeast *Saccharomyces cerevisiae* [21] where the unconstrained model could be fitted appropriately in AT-rich regions while the statistical helix model provides better fits in GC-rich regions [4].

Furthermore, the statistical helix organization, and its underlying dynamics, seems to be finely tuned according to the cell-type. Indeed, chromatin appears to be less constrained in mESC than in mouse liver cells (the statistical helix is more “elongated” in mESC) (Table 1, upper part). This finding is in agreement with several pieces of evidence indicating that, in mESC, chromatin is characterized by an abundance of active chromatin marks [30, 31] and that it displays less compact heterochromatin domains [30, 32, 33]. Therefore, the configuration of the genome makes it more accessible in mESC than in differentiated cells. It is assumed that this specific chromatin organization is essential to establish pluripotency by maintaining the genome in an open, readily accessible state, allowing for maximum plasticity [16].

“Virtual 3C” profiles reconstructed from 5C data obtained in mESC [10] also shows the presence of a very significant modulation in contact frequencies in a 572 kb gene-poor region displaying no apparent locus-specific interaction (chrX:102,338,477-102,910,171) (Additional file 4). Interestingly, here again, the statistical helix model fits better to these data (*R*^*2*^ = 0.52) than the unconstrained chromatin model (*R*^*2*^ = 0.40). Therefore, 5C, as well as quantitative 3C data (Fig. 2), are able to evidence, in mESC, a long-range modulation in contact frequencies which is best described by the statistical helix model.

As previously indicated [4], the existence of a modulation in contact frequencies has important functional implications, at least in the gene-rich TADs where it is prominent. Indeed, locus-specific functional interactions in these TADs necessarily occur from this underlying dynamics of the chromatin. Therefore, any constraints favouring intrinsically the probability of contact between two genomic regions will also favour the probability of interaction between the regulatory elements that they contain. Long-range interactions should thus tend to occur at preferred relative separation distances where the probability of contact is the highest. We previously showed that, in loci containing co-expressed genes, conserved elements (UCSC database) are overrepresented at a distance of ~100 kb from the surrounding Transcriptional Start Sites (TSS) [4]. In the same line, ChIP-seq experiments at 885 loci containing genes overexpressed in the mouse forebrain showed that p300 peaks linked to enhancer activities are more significantly enriched for separation distances of about 70 to 80 kb from the nearest TSS [34]. Finally, extensive 5C experiments focusing on the ENCODE pilot project regions (representing 1 % of the human genome) have recently shown that long-range interactions between TSS and distal elements display a marked asymmetry with a bias for interactions with elements located about 120 kb upstream of the TSS [35]. Altogether, these observations are in agreement with the existence of a long-range (~100 kb) modulation of contact frequencies in gene-rich-TADs, suggesting that the constraints governing statistical helix organization underlie higher-order chromatin dynamics of a very significant part of the genome.

### Simple polymer-physics principles govern chromatin dynamics within TADs

In addition to polymer models as those used in the present work, several other physical models, like the equilibrium and crumpled/fractal globule models, have been developed to describe chromatin dynamics *in vivo* [20]. Crumpled globule features are characteristic of chromatin dynamics above 1 Mb (chromosome territory/inter-TADs dynamics) [8]. However, at that scale, simple polymer-physic models, like the “strings and binders switch” (SBS) model [36], can also reproduce crumpled globule conformations, and finally globule features of chromatin organization within TADs remain unexplored. Using quantitative 3C data (Fig. 2), we showed that, in the absence of any strong locus-specific interaction, contact profiles obtained in gene-rich TADs (Fig. 1) follow a power-law scaling associated to a slope of −1/2. A similar value has been described for mitotic chromosomes for separation distances encompassing 40 kb to 10 Mb [12]. However, our samples are devoid of mitotic chromosomes (interphasic nucleus preparations) and therefore, as previously suggested for distances shorter than 100 kb [8], the contact profiles observed in gene-rich TADs are incompatible with the equilibrium or crumpled globule models. In contrast, they are in good agreement with a more compact conformation as suggested by the SBS model [36]. Therefore, our work reinforce the idea that simple polymer-physics models of chromatin are sufficient to describe chromatin dynamics *in vivo* [4, 37] and it shows that such models and principles also apply within TADs both in mammals and in the fly *Drosophila melanogaster*. Importantly, neither the equilibrium or crumpled globule models nor the “unconstrained chromatin” model, or so far any other known globule or polymer models, including the SBS model, are able to describe the discrete modulation in contact frequencies that we consistently observed within mammalian TADs in diverse experimental and cellular contexts (3C data in Fig. 2; 5C data in Additional file 4; [4]). Only the statistical helix model is able to account for this feature and it is thus, so far, the simplest model to accurately describe the fundamental chromatin dynamics observed within mammalian TADs. However, this model is clearly not sufficient to describe chromatin dynamics when significant locus-specific interactions take place and, in such conditions, more complex polymer models may indeed be required, taking into account chromatin contacts with nuclear compartments and/or attachment of diffusible factors to binding sites on the chromatin [37].

Finally, while the existence of modulated contact frequencies has important implications for chromatin dynamics in a cell population, its interpretation as a helical organization may be far from the reality of an individual conformation at a given time in a single cell. One can note, however, that this model may also be valid to describe chromatin dynamics at the single cell level if the ergodicity of the fluctuations could be verified (*i.e.* if the average fluctuations observed at a given time in a cell population can recapitulate the average fluctuations over time of an individual conformation).

## Conclusion

Two general types of constraints could contribute to the emergence of the statistical helix organization frequencies within mammalian TADs: “bottom-up” constraints, inherent to some intrinsic constituents of the chromatin, or “top-down” constraints imposed by higher-order superstructures, like chromosome territories and TADs. Despite its remarkable impact on chromatin flexibility in gene-rich TADs, histone H1 depletion does not significantly affects statistical helix parameters in mESC (Fig. 3; Table 1, lower part). This indicates that chromatin dynamics at the nucleosomal and supranucleosomal scales could be somewhat uncoupled, suggesting that the constraints imposed on higher-order chromatin dynamics during the interphase may not necessarily rely on intrinsic factors of the chromatin that would affect its nucleosomal organization (“bottom-up” constraints).

Hi-C data have shown that contact frequencies across TAD borders are extremely low [7]. The statistical helix organization observed in mammals is thus necessarily confined within TADs and cannot extend throughout TAD borders. It is therefore tempting to speculate that, in mammals, TADs borders may represent “top-down” constraints impacting chromatin dynamics at higher-order levels by restricting the space that the chromatin could possibly explore at that scale, thus contributing to the emergence of the statistical helix organization. However, this hypothesis is challenged by the fact that no such constraints are observed in *Drosophila* TADs.

How to explain such a difference between these two organisms? Rather than speculating that genome organization principles are intrinsically different (which would appear unlikely for two metazoans), it seems more realistic to postulate that the underlying organization principles are similar, but that constraints applied to higher-order chromatin dynamics have different impacts because of distinct critical features of TAD organization in these two organisms. Indeed, *Drosophila* TADs display a median size of 70 kb [6] which is considerably smaller than that of mammalian TADs. With a median size of more than 800 kb [7], mammalian TADs are more prone to constraints that impact chromatin dynamics at higher-order levels *i.e.* over large genomic distances. Therefore, we propose that, beyond locus-specific interactions, higher-order chromatin dynamics in higher eukaryotes may also rely on “top-down” constraints whose effects are depending on the exact size and organization of the TADs.

## Methods

### Mouse breeding

All experimental designs and procedures are in agreement with the guidelines of the animal ethics committee of the French “Ministère de l’Agriculture” (European directive 2010/63/EU).

### Cell culture

mESC were cultured in serum/LIF conditions as previously described [22].

### Quantitative 3C / SybGreen assays

3C assays were performed from nucleus preparations as previously described [3, 38, 39]. 3C products were quantified (during the linear amplification phase) on a LighCycler 480 II apparatus (Roche) (10 min. at 95 °C followed by 45 cycles 10 s. at 95 °C/8 s. at 69 °C/14 s. at 72 °C) using the Hot-Start Platinum® Taq DNA Polymerase from *Invitrogen* (10966–034), the GoTaq® Hot-Start Polymerase from *Promega* (M5005) and a standard 10X qPCR mix [40] where the usual 300 μM dNTP have been replaced by 1500 μM of CleanAmp dNTP (*Tebu-bio* 040 N-9501-10). Standards curves for qPCR have been generated from BACs (RP serie from *Invitrogen*) as previously described [4]: RP23 55I2 for the *Usp22* locus; RP23 117C15 for the *Dlk1* locus; RP23 463 J10 and RP23 331E7 for the *Lnp* locus; RP23 117 N21 for the *Mtx2* locus; RP23 131E7 for the *Emb* locus; RP23 30H4 and RP23 247C7 for the 3qH2 and 19qC2 gene-poor regions respectively; and a sub-clone derived from RP23 3D5 for the 11qA5 gene-desert region (also see Additional file 1a). Quantitative 3C primers sequences are given in Additional file 6. Data obtained from these experiments are included in Additional file 7 (WT mESC) and Additional file 8 (H1 TKO mESC). The number of sites analysed in each experiment were as follows (Additional file 1b). For WT mESC: *Usp22* locus, for anchor sites F1 and F7, 33 and 35 sites were analysed respectively; *Dlk1* locus, for anchor sites F3/F5/F14 and F16, 9/16/21 and 26 sites were analysed respectively; *Emb* locus, for anchor sites R4 and R7, 30 sites were analysed for each anchor; *Lnp* locus, for anchor site R35, 49 sites were analysed; *Mtx2* locus, for anchor sites R2 and R56, 52 and 50 sites were analysed respectively; 3qH2 gene-poor locus, for anchor sites R6 and R27, 25 sites were analysed for each anchor; 19qC2 gene-poor locus, for anchor sites R41 and R59, 33 sites were analysed for each anchor, and for the 11qA5 gene-desert locus, for anchor sites F5/F25/F35 and F48, 21/20/21 and 20 sites were analysed respectively. For H1 TKO mESC: *Usp22* locus, for anchor sites F1 and F7, 33 and 34 sites were analysed respectively; *Dlk1* locus, for anchor sites F3/F5/F14 and F16, 9/16/21 and 24 sites were analysed respectively; *Emb* locus, for anchor sites R4 and R7, 29 and 30 sites were analysed respectively; *Lnp* locus, for anchor site R35, 49 sites were analysed; *Mtx2* locus, for anchor sites R2 and R56, 52 and 49 sites were analysed respectively; 3qH2 gene-poor locus, for anchor sites R6 and R27, 25 sites were analysed for each anchor; 19qC2 gene-poor locus, for anchor sites R41 and R59, 33 sites were analysed for each anchor, and for the 11qA5 gene-desert locus, for anchor sites F5/F25/F35 and F48, 18/20/21 and 19 sites were analysed respectively.

### Supranucleosomal domains

The supranucleosomal domains (D.I to D.VI) encompass separation distances where random collision frequencies are alternatively lower and higher; They were assessed by statistical analyses (Mann–Whitney U tests) performed on data shown in Figs. 2 and 3. For gene-rich and gene-poor loci : 0 to 35 kb (domain I), 35-70 kb (domain II), 70-115 kb (domain III), 115-160 kb (domain IV), 160-205 kb (domain V) and 205–250 kb (domain VI). For the gene-desert region : 0 to 25 kb (domain I), 25-50 kb (domain II), 50-75 kb (domain III), 75-100 kb (domain IV), 100-125 kb (domain V) and 125–150 kb (domain VI).

### Mathematical methods

We used a model that combines the Freely Jointed Chain/Kratky-Porod worm-like chain models as described in reference [41]. This combined model (equation 3 of reference [21]), which expresses the relation between the cross-linking frequency *X*(*s*) (in mol x liter^−1^ x nm^3^) and the site separation *s* (in kb) along the genome, is as follows:

1 $$ \mathit{\mathsf{X}}\left(\mathit{\mathsf{s}}\right)=\left[K\times \mathsf{0.53}\times {\beta}^{-\raisebox{1ex}{$\mathsf{3}$}\!\left/ \!\raisebox{-1ex}{$\mathsf{2}$}\right.}\times \exp \left(-\raisebox{1ex}{$\mathsf{2}$}\!\left/ \!\raisebox{-1ex}{${\beta}^{\mathsf{2}}$}\right.\right)\times {\left(L\times S\right)}^{-\mathsf{3}}\right] $$

with, for an unconstrained polymer:

2 $$ \beta =\raisebox{1ex}{$s$}\!\left/ \!\raisebox{-1ex}{$S$}\right.\ \left(\mathrm{unconstrained}\ \mathrm{chromatin}\ \mathrm{model}\right) $$

In equation [1], the linear mass density *L* is the length of the chromatin in nm that contains 1 kb of genomic DNA. We used different *L* values estimated from a packing ratio of 6 nucleosomes per 11 nm of chromatin in solution at physiological salt concentrations [42, 43] and a nucleosome repeat length (NRL) of 194 base pairs as found in mouse liver [44] or NRL = 189 and 174 nt for wild-type and TKO mESC respectively [22]. This led to values of *L* = 9.45 nm/kb for mouse liver cells, *L* = 9.70 nm/kb for mESC and *L* = 10.53 nm/kb for TKO mESC. *S* is the length of the Kuhn’s statistical segment in kb, which is a measure for the flexibility of the chromatin. The parameter *K* represents the efficiency of cross-linking which reflects experimental variations [1].

We previously showed that mammalian chromatin undergoes constraints that results in a modulation of contact frequencies along some regions of the chromatin [4]. This modulation can be described by a specific polymer model, called the statistical helix model, where the following *β* term is used in equation [1] (see ref. [4]):

3 $$ \beta =\frac{\sqrt{D^2\times { \sin}^2\left[\frac{\pi \times L\times s}{\sqrt{\pi^2\times {D}^2+{P}^2}}\right]+\left[\frac{P^2\times {L}^2\times {s}^2}{\pi^2\times {D}^2+{P}^2}\right]}}{L\times S}\ \left(\mathrm{statistical}\ \mathrm{helix}\ \mathrm{model}\right) $$

where *P* is the mean Pitch and *D* the mean diameter in nm of the statistical helix. The length of one turn on the statistical helix *Sh* in kb (Table 1) was calculated using best-fit parameters and equation [4]:

4 $$ Sh = \frac{\sqrt{{\left(\pi \times D\right)}^2+\left({P}^2\right)}}{L}\ \left(\mathrm{kb}\right) $$

### Best-fit analyses of quantitative 3C data from mouse ECS

Best-fit analyses were implemented under the R software (R Development Core Team 2008, http://www.R-project.org), as previously described [4]. We used the “*nls* object” (package *stats* version 2.8.1) which determines the nonlinear (weighted) least-squares estimates of the parameters of nonlinear models.

### Best-fit analyses of “Virtual 3C” in the *Drosophila melanogaster*

Hi-C data were obtained from total *Drosophila* embryos and normalized tag numbers were assembled into 5 kb bins as previously described [6, 28]. Datasets have been submitted to Gene Expression Omnibus (GEO) under accession no [GSE61471] (http://www.ncbi.nlm.nih.gov/geo/query/acc.cgi?acc=GSE61471). The relative contact frequencies used to construct the “virtual 3C” profiles were obtained by assembling these 5 kb bins into larger 25 kb (5*5 kb) bins that were analyzed with a step of 5 kb along the chromosomes. For each 25 kb bins, the relative contact frequencies were calculated each 5 kb within a region surrounding 400 kb (80*5 kb bins) from the start of the 25 kb bin. For each “virtual 3C”, the unconstrained chromatin model [eqs. 1 and 2] was fitted to the first 70 kb (14*5 kb bins) using the “*nls2* object” under the R software (R Development Core Team 2008, http://www.R-project.org), and the best-fit parameters were extracted. Statistical analyses of these parameters were performed separately on each chromosome and according to the type of epigenetic domain (Fig. 2 and Additional file 2). Wilcox *p*-values were calculated to assess the significance of differences observed between the values obtained in each case (Additional files 1 and 9).

### Availability of supporting data

The data set supporting the results of this article is available in the Gene Expression Omnibus repository, [GSE61471, http://www.ncbi.nlm.nih.gov/geo/query/acc.cgi?acc=GSE61471].

## Additional files

Additional file 1:
**Genomic maps of the TADs investigated in the present study.** (a) Map of the TADs containing the loci analyzed in mESC. The Hi-C data (http://yuelab.org/hi-c/database.php) ([1]) are displayed on the top of each map. Gene locations are presented as visualized in the UCSC browser. The black squares in the Hi-C data and the location of the BACs (black bars below the genes) help to demarcate the regions analyzed in our quantitative 3C experiments. Red and green rectangles indicate a negative or a positive directionality index respectively, as defined in ref. [1]. Blue rectangles are located at the borders of each TAD. (b) Detailed map of the loci investigated by quantitative 3C. Genes are indicated by full boxes and promoters by thick black arrows above these boxes. The scale-bar indicates the size of 10 kb of sequence. The names of the loci and chromosomal location are indicated above each map. The HindIII (*Usp22*, *Emb*, *Lnp*, *Mtx2,* 19qC2, 3qH2 and 11qA5 loci) or EcoRI (*Dlk1* locus) sites investigated are indicated on the maps. Arrows labeled with a “F” (forward) or a “R” (reverse) indicate the positions of the primers used as anchors in quantitative 3C experiments. The location (mm9), size (in Mb) and gene density (TSS/Mb) of each TAD investigated are indicated on the right. Note that the very low contact frequencies observed for regions investigated on chromosomes 3 and 11qA5 impair the accurate location of TAD borders. TAD sizes provided here are those determined from data published in ref. [1] (Additional file 9). (PDF 300 kb) Additional file 2:
**Nine examples of fits of the unconstrained chromatin model [eqs.** 1 **and** 2 **] on « Virtual 3C » data obtained on** ***Drosophila melanogaster*** **chromosome 2 L.** Because of the small size of TADs in the *Drosophila*, the unconstrained chromatin model was fitted to the first 70 kb of the data. Note that the fit is generally in very good agreement with the unconstrained chromatin model even for larger separation distances. (PDF 250 kb) Additional file 3:
**Epigenetic landscapes and chromatin dynamics of the** ***Drosophila chromosomes.*** “Virtual 3C” were obtained and analysed from each chromosome as described in Fig. 4. Statistical analyses of best-fit parameters were performed separately according to the epigenetic domains (Note that chromosome 4 is exclusively composed of D4 Domains, while chromosome 3 is devoid of such domains). Box-plots show the results obtained for each type of domains on each chromosome. Chromosome X was excluded from the analyses because dosage compensation affects epigenetic landscapes of this chromosome in males and the embryos used in the Hi-C experiments were not sexed. The number of best-fits (*n*) performed in each domain is as follows: for chromosome 2R, D1: *n* = 1391; D2: *n* = 1943; D3: *n* = 350; D4: *n* = 290, for chromosome 3 L, D1: *n* = 1096; D2: *n* = 2650; D3: *n* = 609; D4: *n* = 272, for chromosome 3R, D1: *n* = 1525; D2: *n* = 3197; D3: *n* = 755, for chromosome 4, D4: *n* = 225. (PDF 260 kb) Additional file 4:
**fitting the statistical helix model to contact frequencies quantified by 5C experiments in mECS: (a) 5C Matrix from data obtained in mESC [** 2 **] indicating the 572 kb gene-poor region (region 1) with no apparent locus-specific interaction (chrX:102,338,477-102,910,171) that we used to fit polymer models.** (b) “Virtual 3C” profiles were reconstructed from region 1 and data were compiled in a single graph. Error bars are standard error of the mean of two 5C experiments. Dashed lines delimit supranucleosomal domains that encompass separation distances where contact frequencies are alternatively lower and higher (see Methods). The graph shows the best fit analyses obtained with the unconstrained chromatin model [eqs. 1 and 2] (black curve) or the statistical helix model [eqs. 1 and 3] (red curve). Correlation coefficients (*R*2) are indicated on the graph. For each supranucleosomal domains, the mean contact frequencies and the number (*n*) of experimental points are indicated on the graph. *p*-values (Mann–Whitney *U*-test) account for the significance of the differences observed between the experimental means of two adjacent domains. (PDF 200 kb) Additional file 5:
**Statistical tests.** This table gives, for each chromosome and each domain, the Wilcox *p*-values for the differences observed between the median values of the three parameters presented in Table 2 (*k* = crosslinking efficiency; *L* = compaction; *S* = flexibility). (PDF 72 kb) Additional file 6:
**Quantitative 3C primer sequences.** (XLS 56 kb) Additional file 7:
**Quantitative 3C dataset for WT mouse embryonic stem cells.** (XLS 89 kb) Additional file 8:
**Quantitative 3C dataset for H1 TKO mouse embryonic stem cells.** (XLS 86 kb) Additional file 9:
**Additional references.** (PDF 108 kb)

## Abbreviations

TAD: Topologically Associating Domains
3C: Chromosome Conformation Capture
mESC: mouse Embryonic Stem Cells
H1 TKO: Triple Knock-Out of Histone H1 genes
qPCR: Real-time quantitative polymerase chain reaction
