# Supplementary material for: Distinct polymer physics principles govern chromatin dynamics in mouse and Drosophila topological domains
Source: BMC Genomics. 2015 Aug 15;16(1):607. doi: 10.1186/s12864-015-1786-8 (PMC4536789; doi:10.1186/s12864-015-1786-8)
Supplement: Additional file 1: — Genomic maps of the TADs investigated in the present study. (a) Map of the TADs containing the loci analyzed in mESC. The Hi-C data (http://yuelab.org/hi-c/database.php) ([1]) are displayed on the top of each map. Gene locations are presented as visualized in the UCSC browser. The black squares in the Hi-C data and the location of the BACs (black bars below the genes) help to demarcate the regions analyzed in our quantitative 3C experiments. Red and green rectangles indicate a negative or a positive directionality index respectively, as defined in ref. [1]. Blue rectangles are located at the borders of each TAD. (b) Detailed map of the loci investigated by quantitative 3C. Genes are indicated by full boxes and promoters by thick black arrows above these boxes. The scale-bar indicates the size of 10 kb of sequence. The names of the loci and chromosomal location are indicated above each map. The HindIII (Usp22, Emb, Lnp, Mtx2, 19qC2, 3qH2 and 11qA5 loci) or EcoRI (Dlk1 locus) sites investigated are indicated on the maps. Arrows labeled with a “F” (forward) or a “R” (reverse) indicate the positions of the primers used as anchors in quantitative 3C experiments. The location (mm9), size (in Mb) and gene density (TSS/Mb) of each TAD investigated are indicated on the right. Note that the very low contact frequencies observed for regions investigated on chromosomes 3 and 11qA5 impair the accurate location of TAD borders. TAD sizes provided here are those determined from data published in ref. [1] (Additional file 9). (PDF 300 kb) [file 12864_2015_1786_MOESM1_ESM.pdf]

Additional file 1a

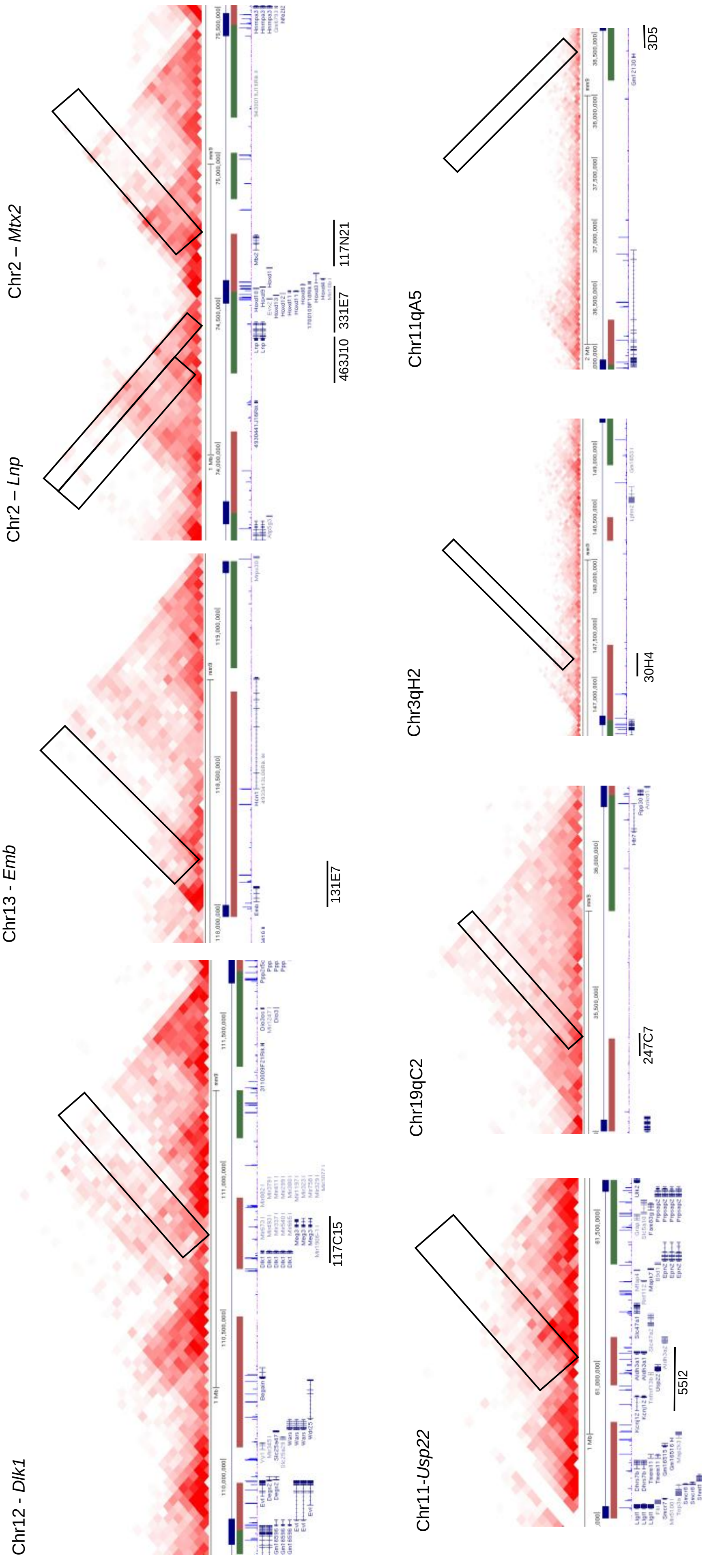

Additional file 1b

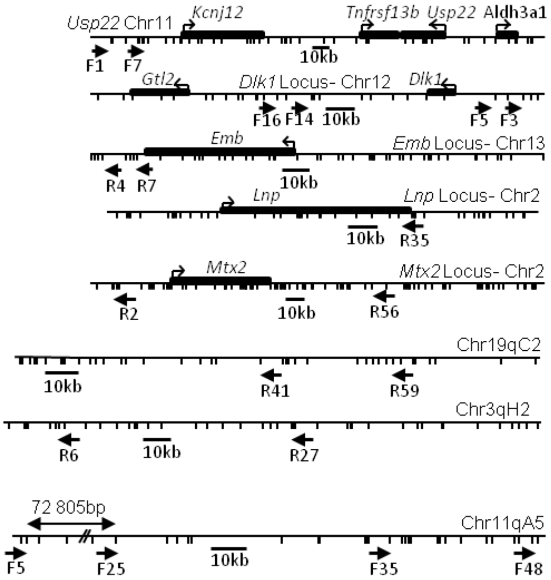

TAD Position (mm9)	Size (Mb)	RefSeq #	RefSeq TSS/Mb	Genomic context
Chr11: 60,480,000-61,560,000	1.08	27	25	Gene-rich
Chr12: 109,760,000-111,640,000	1.88	18	9.6	
Chr13: 117,960,000-119,160,000	1.20	3	2.5	
Chr2: 73,760,000-74,520,000	0.76	6	7.9	
Chr2: 74,520,000-75,440,000	0.92	9	9.8	
Chr19: 35,040,000-36,840,000	1.80	4	2.2*	Gene-poor
Chr3: 146,640,000-149,200,000	2.56	2	0.78	
Chr11: 35,840,000-38,560,000	2.72	1	0.37	Gene-desert

* This TAD possesses several genes near its borders. However, the gene-poor locus investigated is located far-away from them, in the center of the TAD (see Additional file 1a).

Additional file 1 : Genomic maps of the TADs investigated in the present study. (a) Map of the TADs containing the loci analyzed in mESC. The Hi-C data (http://yuelab.org/hi-c/database.php) ([1]) are displayed on the top of each map. Gene locations are presented as visualized in the UCSC browser. The black squares in the Hi-C data and the location of the BACs (black bars below the genes) help to demarcate the regions analyzed in our quantitative 3C experiments. Red and green rectangles indicate a negative or a positive directionality index respectively, as defined in ref. [1]. Blue rectangles are located at the borders of each TAD. **(b)** Detailed map of the loci investigated by quantitative 3C. Genes are indicated by full boxes and promoters by thick black arrows above these boxes. The scale-bar indicates the size of 10kb of sequence. The names of the loci and chromosomal location are indicated above each map. The HindIII (*Usp22*, *Emb*, *Lnp*, *Mtx2*, 19qC2, 3qH2 and 11qA5 loci) or EcoRI (*Dlk1* locus) sites investigated are indicated on the maps. Arrows labeled with a "F" (forward) or a "R" (reverse) indicate the positions of the primers used as anchors in quantitative 3C experiments. The location (mm9), size (in Mb) and gene density (TSS/Mb) of each TAD investigated are indicated on the right. Note that the very low contact frequencies observed for regions investigated on chromosomes 3 and 11qA5 impair the accurate location of TAD borders. TAD sizes provided here are those determined from data published in ref. [1].
