# Supplementary material for: Distinct polymer physics principles govern chromatin dynamics in mouse and Drosophila topological domains
Source: BMC Genomics. 2015 Aug 15;16(1):607. doi: 10.1186/s12864-015-1786-8 (PMC4536789; doi:10.1186/s12864-015-1786-8)
Supplement: Additional file 2: — Nine examples of fits of the unconstrained chromatin model [eqs. 1 and 2 ] on « Virtual 3C » data obtained on Drosophila melanogaster chromosome 2 L. Because of the small size of TADs in the Drosophila, the unconstrained chromatin model was fitted to the first 70 kb of the data. Note that the fit is generally in very good agreement with the unconstrained chromatin model even for larger separation distances. (PDF 250 kb) [file 12864_2015_1786_MOESM2_ESM.pdf]

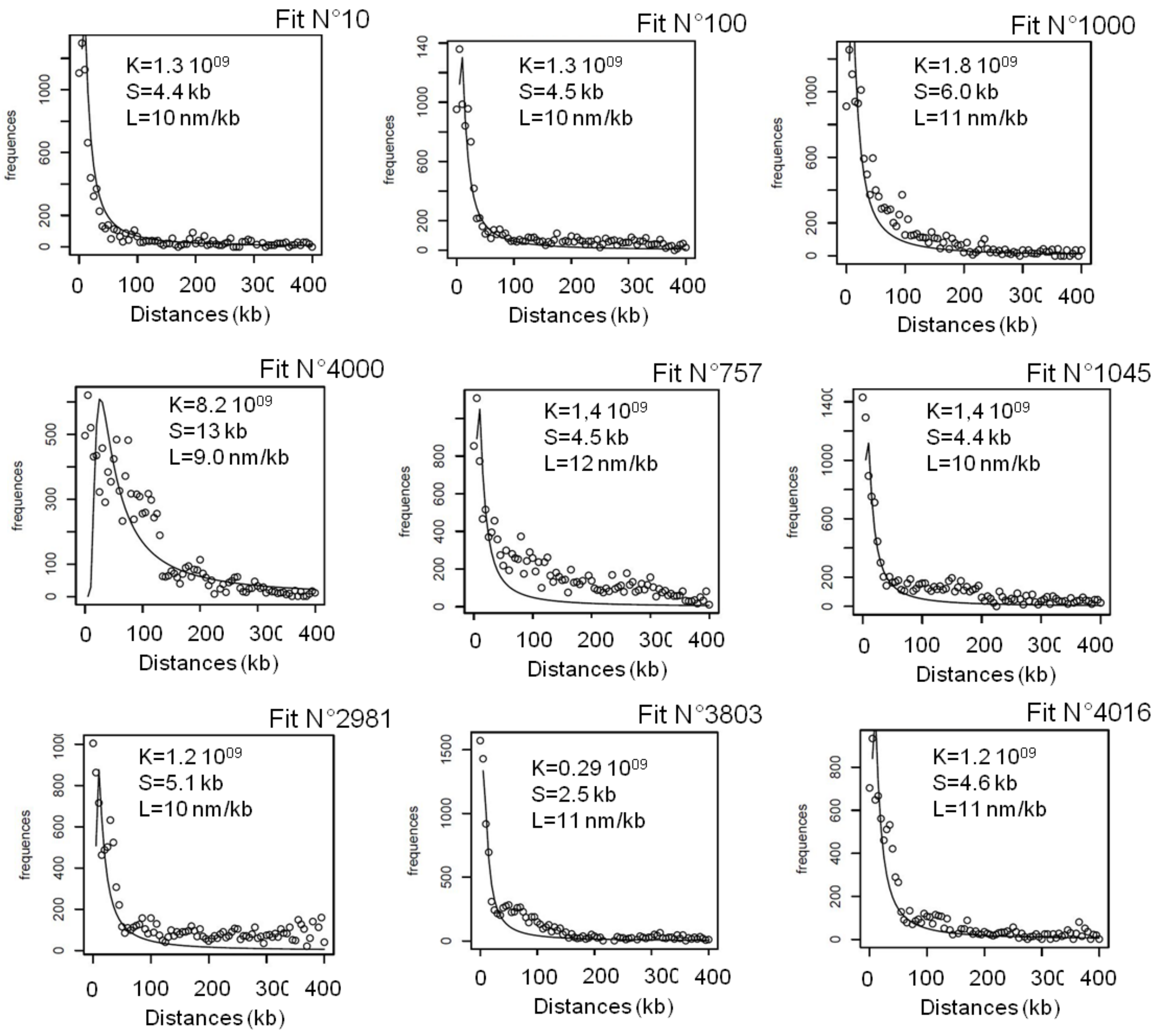

Additional file 2 : Nine examples of fits of the unconstrained chromatin model [eqs. 1 and 2] on « Virtual 3C » data obtained on *Drosophila melanogaster* chromosome 2L. Because of the small size of TADs in the *Drosophila*, the unconstrained chromatin model was fitted to the first 70kb of the data. Note that the fit is generally in very good agreement with the unconstrained chromatin model even for larger separation distances.
