# Supplementary material for: Distinct polymer physics principles govern chromatin dynamics in mouse and Drosophila topological domains
Source: BMC Genomics. 2015 Aug 15;16(1):607. doi: 10.1186/s12864-015-1786-8 (PMC4536789; doi:10.1186/s12864-015-1786-8)
Supplement: Additional file 3: — Epigenetic landscapes and chromatin dynamics of the Drosophila chromosomes. “Virtual 3C” were obtained and analysed from each chromosome as described in Fig. 4. Statistical analyses of best-fit parameters were performed separately according to the epigenetic domains (Note that chromosome 4 is exclusively composed of D4 Domains, while chromosome 3 is devoid of such domains). Box-plots show the results obtained for each type of domains on each chromosome. Chromosome X was excluded from the analyses because dosage compensation affects epigenetic landscapes of this chromosome in males and the embryos used in the Hi-C experiments were not sexed. The number of best-fits (n) performed in each domain is as follows: for chromosome 2R, D1: n = 1391; D2: n = 1943; D3: n = 350; D4: n = 290, for chromosome 3 L, D1: n = 1096; D2: n = 2650; D3: n = 609; D4: n = 272, for chromosome 3R, D1: n = 1525; D2: n = 3197; D3: n = 755, for chromosome 4, D4: n = 225. (PDF 260 kb) [file 12864_2015_1786_MOESM3_ESM.pdf]

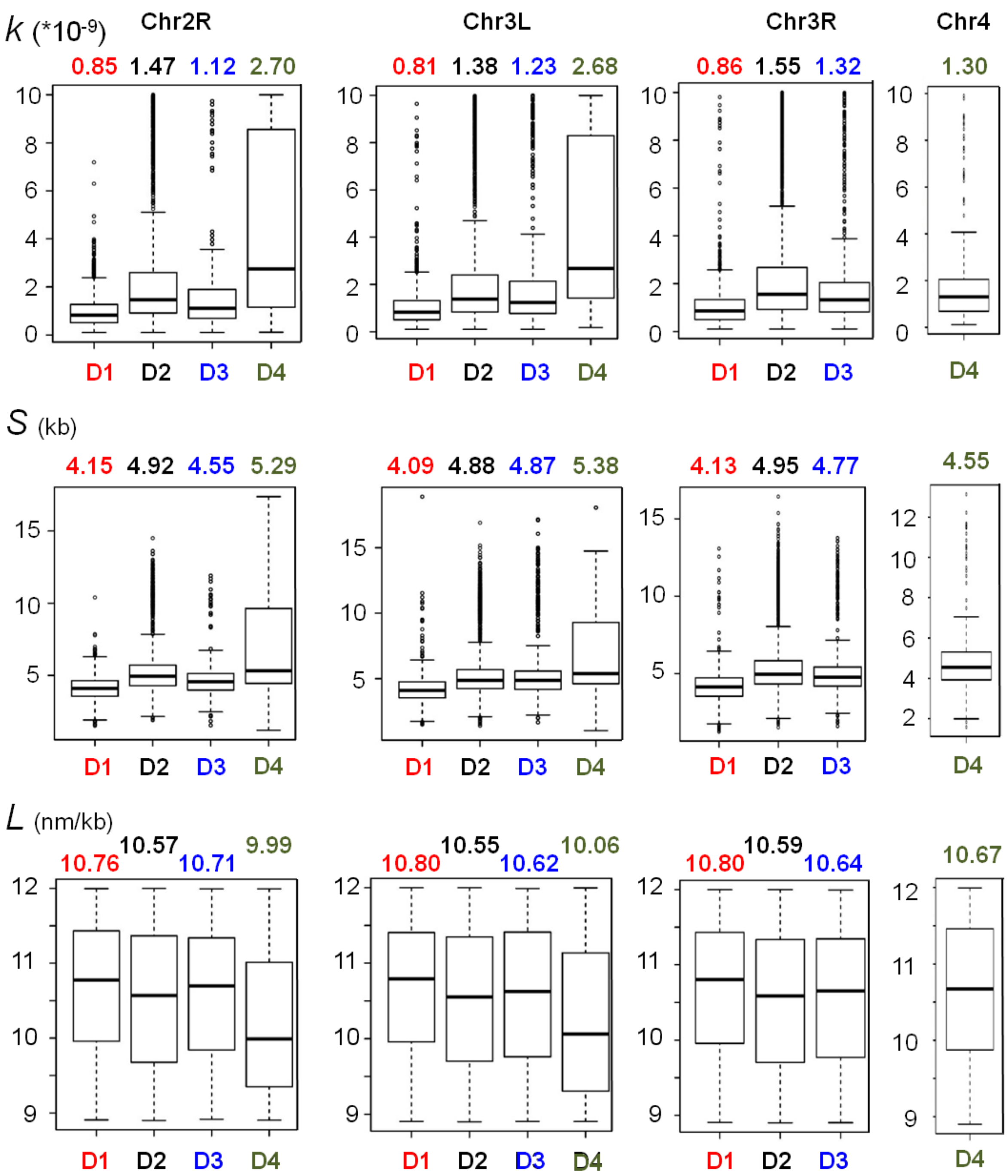

Additional file 3 : Epigenetic landscapes and chromatin dynamics of the *Drosophila* chromosomes. "Virtual 3C" were obtained and analysed from each chromosome as described in Figure 4. Statistical analyses of best-fit parameters were performed separately according to the epigenetic domains (Note that chromosome 4 is exclusively composed of D4 Domains, while chromosome 3 is devoid of such domains). Box-plots show the results obtained for each type of domains on each chromosome. Chromosome X was excluded from the analyses because dosage compensation affects epigenetic landscapes of this chromosome in males and the embryos used in the Hi-C experiments were not sexed. The number of best-fits (n) performed in each domain is as follows: for chromosome 2R, D1: n=1391; D2: n=1943; D3:

n=350; D4: *n*=290, for chromosome 3L, D1: *n*=1096; D2: *n*=2650; D3: *n*=609; D4: *n*=272, for chromosome 3R, D1: *n*=1525; D2: *n*=3197; D3: *n*=755, for chromosome 4, D4: *n*=225.
