# Supplementary material for: Distinct polymer physics principles govern chromatin dynamics in mouse and Drosophila topological domains
Source: BMC Genomics. 2015 Aug 15;16(1):607. doi: 10.1186/s12864-015-1786-8 (PMC4536789; doi:10.1186/s12864-015-1786-8)
Supplement: Additional file 4: — fitting the statistical helix model to contact frequencies quantified by 5C experiments in mECS: (a) 5C Matrix from data obtained in mESC [ 2 ] indicating the 572 kb gene-poor region (region 1) with no apparent locus-specific interaction (chrX:102,338,477-102,910,171) that we used to fit polymer models. (b) “Virtual 3C” profiles were reconstructed from region 1 and data were compiled in a single graph. Error bars are standard error of the mean of two 5C experiments. Dashed lines delimit supranucleosomal domains that encompass separation distances where contact frequencies are alternatively lower and higher (see Methods). The graph shows the best fit analyses obtained with the unconstrained chromatin model [eqs. 1 and 2] (black curve) or the statistical helix model [eqs. 1 and 3] (red curve). Correlation coefficients (R2) are indicated on the graph. For each supranucleosomal domains, the mean contact frequencies and the number (n) of experimental points are indicated on the graph. p-values (Mann–Whitney U-test) account for the significance of the differences observed between the experimental means of two adjacent domains. (PDF 200 kb) [file 12864_2015_1786_MOESM4_ESM.pdf]

A

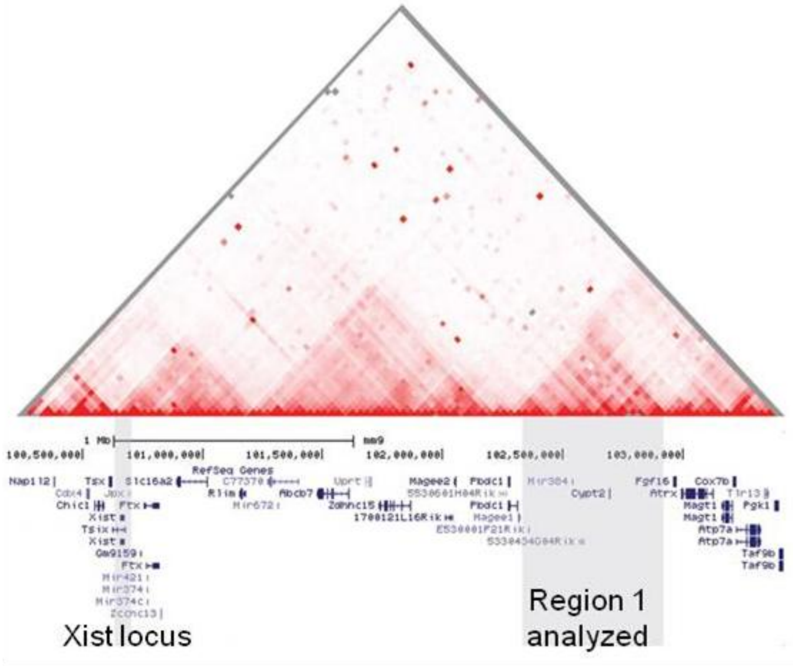
1 Mb
mm9
100,500,000
101,000,000
101,500,000
102,000,000
102,500,000
103,000,000
RefSeq Genes
Xist locus
Region 1
analyzed

B

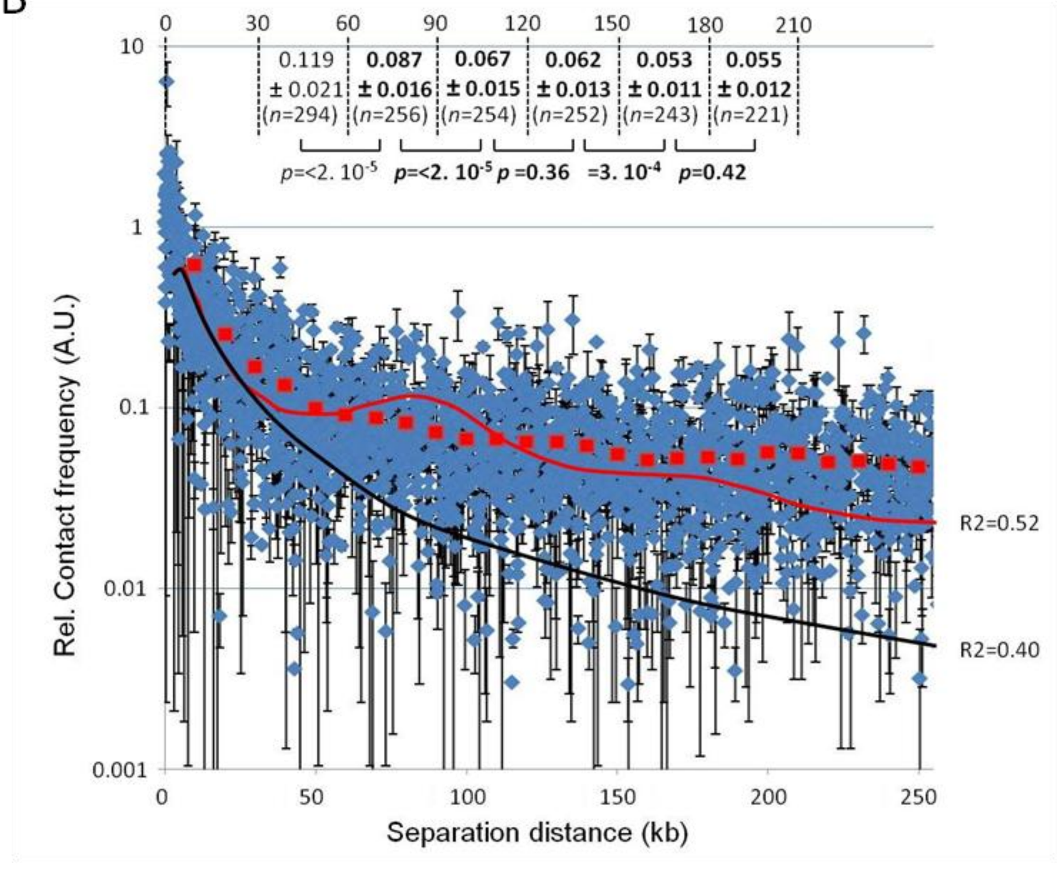
0
30
60
90
120
150
180
210
0.119
± 0.021
(n=294)
0.087
± 0.016
(n=256)
0.067
± 0.015
(n=254)
0.062
± 0.013
(n=252)
0.053
± 0.011
(n=243)
0.055
± 0.012
(n=221)
p=<2. 10-5
p=<2. 10-5
p =0.36
=3. 10-4
p=0.42
10
1
0.1
0.01
0.001
R2=0.52
R2=0.40
Rel. Contact frequency (A.U.)
0
50
100
150
200
250
Separation distance (kb)

Additional file 4 : fitting the statistical helix model to contact frequencies quantified by 5C experiments in mECS: (a) 5C Matrix from data obtained in mESC [2] indicating the 572kb gene-poor region (region 1) with no apparent locus-specific interaction (chrX:102,338,477-102,910,171) that we used to fit polymer models. **(b)** “Virtual 3C” profiles were reconstructed from region 1 and data were compiled in a single graph. Error bars are standard error of the mean of two 5C experiments. Dashed lines delimit supranucleosomal domains that encompass separation distances where contact frequencies are alternatively lower and higher (see *Methods*). The graph shows the best fit analyses obtained with the unconstrained chromatin model [eqs. 1 and 2] (black curve) or the statistical helix model [eqs. 1 and 3] (red curve). Correlation coefficients (R^2) are indicated on the graph. For each supranucleosomal domains, the mean contact frequencies and the number (n) of experimental points are indicated on the graph. p-values (Mann-Whitney U-test) account for the significance of the differences observed between the experimental means of two adjacent domains.
