# Supplementary material for: Distinct polymer physics principles govern chromatin dynamics in mouse and Drosophila topological domains
Source: BMC Genomics. 2015 Aug 15;16(1):607. doi: 10.1186/s12864-015-1786-8 (PMC4536789; doi:10.1186/s12864-015-1786-8)
Supplement: Additional file 5: — Statistical tests. This table gives, for each chromosome and each domain, the Wilcox p-values for the differences observed between the median values of the three parameters presented in Table 2 (k = crosslinking efficiency; L = compaction; S = flexibility). (PDF 72 kb) [file 12864_2015_1786_MOESM5_ESM.pdf]

Additional file 5: Statistical tests. This table gives, for each chromosome and each domain, the Wilcox *p*-values for the differences observed between the median values of the three parameters presented in Table 2 (*k*=crosslinking efficiency; *L*=compaction; *S*=flexibility).

Chr2L

	k				*S* (kb)				*L* (nm/kb)			
pValue	D1	D 2	D 3	D4	D1	D 2	D 3	D4	D1	D 2	D 3	D4
D1	1	$7.80\ 10^{-84}$	$3.33\ 10^{-33}$	$5.27\ 10^{-55}$	1	$2.93\ 10^{-96}$	$6.51\ 10^{-42}$	$6.12\ 10^{-42}$	1	$1.59\ 10^{-04}$	0.0462	$2\ 10^{-04}$
D2	$7.80\ 10^{-84}$	1	0.0237	$3.5\ 10^{-15}$	$2.93\ 10^{-96}$	1	0.197	$1.33\ 10^{-05}$	$1.59\ 10^{-04}$	1	0.498	0.0333
D3	$3.33\ 10^{-33}$	0.0237	1	$6.47\ 10^{-15}$	$6.51\ 10^{-42}$	0.197	1	$1.47\ 10^{-05}$	0.0462	0.498	1	0.0373
D4	$5.27\ 10^{-55}$	$3.5\ 10^{-15}$	$6.47\ 10^{-15}$	1	$6.12\ 10^{-42}$	$1.33\ 10^{-05}$	$1.47\ 10^{-05}$	1	$2\ 10^{-04}$	0.0333	0.0373	1

Chr2R

D1	1	$1.63\ 10^{-109}$	$2.29\ 10^{-14}$	$1.53\ 10^{-60}$	1	$6.08\ 10^{-119}$	$1.08\ 10^{-15}$	$6.62\ 10^{-54}$	1	$2.73\ 10^{-06}$	0.144	$2.9\ 10^{-13}$
D2	$1.63\ 10^{-109}$	1	$2.56\ 10^{-10}$	$1.25\ 10^{-13}$	$6.08\ 10^{-119}$	1	$1.63\ 10^{-12}$	$3.67\ 10^{-07}$	$2.73\ 10^{-06}$	1	0.169	$1.14\ 10^{-06}$
D3	$2.29\ 10^{-14}$	$2.56\ 10^{-10}$	1	$4.11\ 10^{-22}$	$1.08\ 10^{-15}$	$1.63\ 10^{-12}$	1	$1.10\ 10^{-17}$	0.144	0.169	1	$6.13\ 10^{-07}$
D4	$1.53\ 10^{-60}$	$1.25\ 10^{-13}$	$4.11\ 10^{-22}$	1	$6.62\ 10^{-54}$	$3.67\ 10^{-07}$	$1.10\ 10^{-17}$	1	$2.9\ 10^{-13}$	$1.14\ 10^{-06}$	$6.13\ 10^{-07}$	1

Chr3L

D1	1	$1.3\ 10^{-80}$	$1.75\ 10^{-31}$	$5.87\ 10^{-75}$	1	$5.76\ 10^{-98}$	$1.22\ 10^{-48}$	$2.07\ 10^{-58}$	1	$1.39\ 10^{-05}$	0.0176	$2.77\ 10^{-11}$
D2	$1.3\ 10^{-80}$	1	0.0486	$1.93\ 10^{-27}$	$5.76\ 10^{-98}$	1	0.934	$1.69\ 10^{-10}$	$1.39\ 10^{-05}$	1	0.485	$1.11\ 10^{-06}$
D3	$1.75\ 10^{-31}$	0.0486	1	$1.98\ 10^{-24}$	$1.22\ 10^{-48}$	0.934	1	$4.46\ 10^{-08}$	0.0176	0.485	1	$7.54\ 10^{-06}$
D4	$5.87\ 10^{-75}$	$1.93\ 10^{-27}$	$1.98\ 10^{-24}$	1	$2.07\ 10^{-58}$	$1.69\ 10^{-10}$	$4.46\ 10^{-08}$	1	$2.77\ 10^{-11}$	$1.11\ 10^{-06}$	$7.54\ 10^{-06}$	1

Chr3R

D1	1	$5.90\ 10^{-143}$	$1.48\ 10^{-41}$	1	$5.21\ 10^{-158}$	$3.6\ 10^{-52}$	1	$4.06\ 10^{-08}$	0.00485
D2	$5.90\ 10^{-143}$	1	$1.59\ 10^{-08}$	$5.21\ 10^{-158}$	1	$8\ 10^{-07}$	$4.06\ 10^{-08}$	1	0.268
D3	$1.48\ 10^{-41}$	$1.59\ 10^{-08}$	1	$3.6\ 10^{-52}$	$8\ 10^{-07}$	1	0.00485	0.268	1
