# Supplementary material for: Distinct polymer physics principles govern chromatin dynamics in mouse and Drosophila topological domains
Source: BMC Genomics. 2015 Aug 15;16(1):607. doi: 10.1186/s12864-015-1786-8 (PMC4536789; doi:10.1186/s12864-015-1786-8)
Supplement: Additional file 9: — Additional references. (PDF 108 kb) [file 12864_2015_1786_MOESM9_ESM.pdf]

Additional file 9 - Additional references.

These references are cited in the additional data files.

1. Dixon JR, Selvaraj S, Yue F, Kim A, Li Y, Shen Y, Hu M, Liu JS, Ren B: **Topological domains in mammalian genomes identified by analysis of chromatin interactions**. *Nature* 2012, **485**(7398):376-380.
2. Nora EP, Lajoie BR, Schulz EG, Giorgetti L, Okamoto I, Servant N, Piolot T, van Berkum NL, Meisig J, Sedat J, Gribnau J, Barillot E, Bluthgen N, Dekker J, Heard E: **Spatial partitioning of the regulatory landscape of the X-inactivation centre**. *Nature* 2012, **485**(7398):381-385. .
